# Exosomes derived from M1 macrophages aggravate neointimal hyperplasia following carotid artery injuries in mice through miR-222/CDKN1B/CDKN1C pathway

**DOI:** 10.1038/s41419-019-1667-1

**Published:** 2019-05-29

**Authors:** Zeng Wang, Hong Zhu, Hongtao Shi, Huan Zhao, Rifeng Gao, Xinyu Weng, Rongle Liu, Xiao Li, Yunzeng Zou, Kai Hu, Aijun Sun, Junbo Ge

**Affiliations:** 1 Institute of Biomedical Sciences, Fudan University, 200032 Shanghai, China; 2 Department of Cardiology, Zhongshan Hospital, Fudan University. Shanghai Institute of Cardiovascular Diseases, Zhongshan Hospital, Fudan University, 200032 Shanghai, PR China; 3 Department of Pathology, LiShui Central Hospital, The Fifth Affiliated Hospital of Wenzhou Medical College, ZheJiang, China; 4 Department of Cardiovascular Medicine, The Second Affiliated Hospital of Nanchang University, Nanchang, China

**Keywords:** Cell invasion, Carotid artery disease

## Abstract

The role of M1 macrophages (M1M)-derived exosomes in the progression of neointimal hyperplasia remains unclear now. Using a transwell co-culture system, we demonstrated that M1M contributed to functional change of vascular smooth muscle cell (VSMC). We further stimulated VSMCs with exosomes isolated from M1M. Our results demonstrated that these exosomes could be taken up by VSMCs through macropinocytosis. Using a microRNA array assay, we identified that miR-222 originated from M1M-derived exosomes triggered the functional changes of VSMCs. In addition, we confirmed that miR-222 played a key role in promoting VSMCs proliferation and migration by targeting Cyclin Dependent Kinase Inhibitor 1B (CDKN1B) and Cyclin Dependent Kinase Inhibitor 1C (CDKN1C) in vitro. In vivo, M1M-derived exosomes significantly aggravated neointima formation following carotid artery ligation injury and wire injury and these effects were partly abolished by miR-222 inhibitor 2′OMe-miR-222. Our findings thus suggest that exosomes derived from M1M could aggravate neointimal hyperplasia through delivering miR-222 into VSMCs. Future studies are warranted to validate if the post-injury vascular neointimal hyperplasia and restenosis could be attenuated by inhibiting miR-222.

## Introduction

Percutaneous coronary interventions (PCI) have become the first choice for the treatment of acute coronary syndromes, but patients undergoing PCI face increased risk of restenosis^1^. Our understanding on the pathophysiological mechanisms of the proliferation and migration of smooth muscle cells as well as neointimal hyperplasia, which is the major driving force of restenosis, has improved greatly through continuous scientific researches. However, reports on the interaction and communication between inflammatory and smooth muscle cells are scanty^2,3^. It is known that macrophages will accumulate around the injured area after vascular injury, thereby promote neointimal hyperplasia or even atherosclerotic plaque formation through releasing cellular secretions^4–6^.

Exosomes, a subset of extracellular vesicles, are secreted by all kinds of cells in diameter of 30–150 nm. For a long time, the exosomes were considered as residues of cells, until some studies found that the exosomes could play an important role in intercellular communication by transferring proteins, lipids, and microRNAs^7,8^. MicroRNA is a class of endogenous, small, and non-coding RNAs, which can negatively regulate the expression of target genes by binding to its mRNA^9,10^. Therefore, microRNA can be used not only as a biomarker of diagnostics, but also as a regulator of numerous biological functions^11^. Several miRNAs such as miR-21, miR-126, miR-155, and miR-222 have been reported to play crucial roles in neointima formation and vascular remodeling^10,12–15^. It is known that miR-222 could promote the migration and proliferation of smooth muscle cells and is upregulated in neointima after balloon injury, but the origin of miR-222 is still an issue of debate^9,10,16^. Several target genes of miR-222 are found to be implicated in the development of vascular remodeling such as CDKN1B and CDKN1C^17,18^. In this study, we investigated whether and how exosomes derived from M1 macrophages (M1M) exert their role on carotid artery vascular remodeling, focusing on the mediating role of miR-222.

## Materials and methods

### Animal models

Mice were firstly anaesthetized by 40 mg/kg sodium pentobarbital (Sigma–Aldrich, St Lois, MO, USA) via intraperitoneal injection. The extent of anaesthesia was assessed by mouse’s reaction to the toe pinching during the splenectomy or the carotid artery injury surgery. Wire injury of the mouse carotid artery was performed as described previously^19^. The left common carotid artery, including bifurcation, was exposed via a midline incision on the ventral side of the neck. The left common carotid artery was dissected and injured by passing a curved flexible wire (0.38-mm diameter, Reference Part Number: C-SF-15–20, Cook Medical European Shared Services, Ireland) three times toward and forth with rotation.

Mouse carotid artery ligation was performed as previously described. The left common carotid arteries were exposed, and the bifurcation of internal/external branches was ligated completely with a 6-0 silk suture.

All mice were maintained at the Zhongshan Experimental Animal Center, Fudan University, Shanghai. The animal experiments were conducted according to the Guide for the Care and Use of Laboratory Animals published by the US National Institutes of Health (NIH publication no. 85-23, revised 1996) and were approved by the Animal Care and Use Committee of Fudan University. All studies involving animals are reported in accordance with the ARRIVE guidelines for reporting experiments involving animals.

### Local exosome delivery into the injured vascular walls

To deliver R1-EXO into the injured vascular tissue and to avoid any potential systemic side effects, we applied an established local delivery model via pluronic gel F-127 as described in previous reports with little modification^9,20–22^. Briefly, immediately after wire injury of the mouse common carotid artery, R1-EXO (10 μg) in PBS or transfection solutions mixed with 2′OMe-miR-222 (10 μg), vehicle (DMEM) were infused into the ligated segment of the common carotid artery for 30 min. Then, 50 μg R1-EXO or oligonucleotides preloaded into 50 μl 20% pluronic gel F-127 (Sigma) at 4 °C was applied locally to the adventitia around injured artery segments. The left carotid and right carotid arteries were harvested 28 days after surgery by perfusion-fixation with formalin at physiological pressure for histological and immunohistochemical analysis.

### Cell culture

Human aortic vascular smooth muscle cells (HA-VSMCs) were cultured in Smooth Muscle Cell Medium (SMCM), supplemented with a mixture of smooth muscle growth factors and fetal bovine serum to a final concentration of 2%. Cells between passages 3 and 7 were used for this study.

Human monocytic cell line THP-1 was cultured and maintained in complete RPMI 1640 (Gibco, Life Technologies), supplemented with 10% (v/v) heat-inactivated fetal bovine serum (FBS) and 1% penicillin/streptomycin. THP-1 cells were differentiated into macrophages by treatment with 100 nM phorbol myristate acetate (PMA; Sigma–Aldrich, Poland) for 24 h, and then the adherent M0 type macrophages were polarized towards M1 macrophages by treatment with 100 ng/mL of Lipopolysaccharide (LPS) and 20 ng/mL of Interferon-γ (IFN-γ) for 48 h.

RAW264.7 was cultured in Dulbecco’s modified Eagle’s medium (DMEM, Gibco, Life Technologies) containing 10% heat-inactivated FBS and 1% penicillin/streptomycin. RAW264.7 macrophages were either treated (M1) or untreated (M0) with 100 ng/mL of Lipopolysaccharide (LPS) and 20 ng/mL of Interferon-γ (IFN-γ) for 48 h. For all experiments, cells were grown at 37 °C in a humidified atmosphere containing 5% (v/v) CO_2_.

### Cell co-culture

For evaluating the effect of M1M on smooth muscle cell proliferation in vitro, a cell co-culture model was established. M1 macrophages incubated in the transwell overnight, and then the transwell was inserted into another 24-well culture plate where smooth muscle cells had been cultured at a density of 2.5 × 10^4^ cells/well for 1 day. The two kinds of cells in the transwell-chambers were co-cultured for 36 h for further experiments.

For evaluating the effect of M1M on smooth muscle cell migration in vitro, a cell co-culture model was established. VSMCs incubated in the transwell overnight, and then the transwell was inserted into another 24-well culture plate where M1 macrophages had been cultured at a density of 4 × 10^4^ cells/well for 1 day. The two kinds of cells in the transwell-chambers were co-cultured for 36 h for further experiments.

To clarify whether exosomes were involved in the process of VSMCs functional change induced by M1 macrophages, GW4869 (Sigma–Aldrich, California, USA) was used at a concentration of 10 μM to reduce the release of exosome from M1 macrophages. Before M1 macrophages were co-cultured with VSMCs, they were stimulated with GW4869 for 8 h.

### Isolation and polarization of mouse bone-marrow-derived macrophages (BMDM)

Bone-marrow-derived macrophages were flushed out from the femur and tibia of 6-week-old mice and differentiated in complete DMEM/F12 medium supplemented with 50 ng/mL macrophage-colony stimulating factor (MCSF). On day 7, BMDM were treated with LPS for 24 h to generate M1 phenotype macrophages.

### Isolation and cultivation of mouse aorta smooth muscle cells (MA-SMC)

MA-SMC were isolated from aortas of 6-week-old mice and digested in enzyme solution at room temperature. Floating cells were harvested and cultured in complete DMEM/F12 medium. Cells between passages 1 and 3 were used for this study.

### Isolation and identification of exosomes

For exosomes secreted by cultured cell lines, conditioned medium (CM) was first prepared by incubating cells in media containing exosome-depleted FBS (prepared by ultracentrifugation at 100,000 × *g* at 4 °C for at least 4 h), and pre-cleared by centrifugation at 500 × *g* for 15 min and then at 10,000 × *g* for 20 min. Exosomes were isolated by ultracentrifugation at 100,000 × *g* for 150 min, and washed in PBS using the same ultracentrifugation conditions. When indicated, DiI (1,1′-Dioctadecyl-3,3,3′,3′-tetramethylindocarbocyanine perchlorate; Sigma) was added into the PBS at 1 μM and incubated for 20 min before the washing spin, followed by an additional wash to remove the excess dye. The pelleted exosomes were resuspended in ~100 µL of PBS, and subjected to further treatments^23^.

Nanosight (Malvern, Malvern, UK) analysis and transmission electron microscopy (TEM) (JEOL JMPEG-PTMC-1230, Japan) were used to identify exosomes. RNA and proteins were extracted from exosomes for further analysis. Protein markers, CD63, Alix, Hsp70 were determined by immunoblotting. The BCA protein assay kit was used to quantify the exosomes. We named T1-EXO or T0-EXO as the exosomes isolated from THP-1 derived M1 or M0 type macrophages. We named R1-EXO or R0-EXO as the exosomes isolated from RAW264.7 derived M1 or M0 type macrophages.

### Cellular uptake and endocytic mechanisms in vitro

HA-VSMC cells were seeded at a density of 2.5 × 10^4^ cells/well in six-well plates, incubated for 12 h, and checked under the microscope for confluency and morphology. After being pre-incubated with Hank’s balanced salt solution (HBSS) for 15 min, HA-VSMC cells were incubated with DiI-labeled T1-EXO at the final concentration from 0 to 5 μg/mL at 37 °C for 6 h.

For cellular uptake mechanism assay, HA-VSMC cells were seeded in six-well plates. After checking the confluency and morphology, inhibit agents including sucrose (0.45 M) and 5-(N,N-dimethyl) amiloride hydrochloride (DMA, 10 m M) were added into each well and incubated for 30 min, respectively. Then the compounds were withdrawn from the wells, and DiI-labeled T1-EXO was added at the final concentration of 2.5 μg/mL. After incubation, the cells were visualized under fluorescent microscope (Leica DMI 4000B, Germany).

### Permeation of EXO into smooth muscle cells

To investigate the permeation efficacy across endothelial cell layer of the T1-EXO in vitro, an endothelial cell monolayer and smooth muscle cell co-culture model was established. The endothelial cells incubated in transwell for 1 day, and then the transwell was inserted into another 24-well culture plate where smooth muscle cells had been cultured overnight. The transwell-chambers were co-cultured for 24 h to establish the co-cultured model. Fluorescence-labeled exoxomes were added into each transwell chamber at a concentration of 5 μg/mL. After 36 h of incubation at 37 °C, the smooth muscle cells were analyzed using an Leica fluorescence microscopy.

### Cell proliferation assay

#### Cell Counting Kit-8

Cell Counting Kit -8 assay was adopted to test the proliferation of HA-VSMC cells in the presence of different doses of T1-EXO. The cells were seeded onto 96-well flat-bottomed plates with a density of 2500 cells/well and then were incubated in 5% CO_2_ atmosphere at 37 °C, followed by samples teatment for different times. After incubation, the medium was added with 10 μL of CCK8 solution. The absorbance (ODs) value was measured at 450 nm using microplate reader (Synergy^TM^ H4; BioTek Instruments, Inc. USA)

#### EdU incorporation assay

DNA synthesis was also analysed using a BeyoClick EdU Apollo488 *In Vitro* Imaging Kit (Beyotime Co., Ltd, Shanghai, China) according to the manufacturer’s instructions.

### Cell migration assay

#### Wound healing

HA-VSMC cells were seeded in six-well plates and cultured until cell monolayers formed. Monolayers were wounded by manual scraping with a 10-μL micropipette tip. The cells were then incubated with serum-free medium supplemented with or without indicated concentrations of exosomes or other factors for 36 h. Wound repair was analysed measuring the injured area covered by cells counted from the wounding borders with the Image J software.

#### Transwell

HA-VSMCs were cultured in FBS-free SMCM for 24 h. An aliquot (2 × 10^4^ cells/200 µl) of cells in serum-free SMCM was dispensed into the transwell inserts (8 µm pore size, Costar, USA) pre-coated with 0.5% gelatin (Sigma, G1393), and total medium with or without T1-EXO was placed in the lower chamber. The transwell plates were incubated at 37 °C in a 5% CO_2_ incubator for 12–36 h. The migrated cells in the bottom side were stained with Crystal Violet dye.

### Lentivirus infection

The mir-222-up lentivirus was purchased from Genechem Co., LTD (Shanghai, China). An empty vector was constructed in the same manner as a negative control. All the experiments were conducted according to the manufacturer’s protocol. HA-VSMCs were 60–80% confluent and cells were washed twice with 1 mL PBS. In our preliminary experiment, different doses of GFP-labeled lentivirus were added into the cells and infection efficiency was determined. The obtained optimal multiplicity of infection (MOI) was used for further lentivirus infection experiments. The infected cells were washed with new culture media for subsequent experiments.

### Real-time q-PCR

Total RNA was extracted using TRIzol reagent (Sangon, Shanghai, China) from cells or exosomes. The purity of the isolated RNA was determined by the optical density 260/280 ratio using the NanoDrop ND-2000 (Thermo Scientific). The isolated RNA was reverse transcribed using the miRNA qRT-PCR Starter Kit (Ribobio, China). RT-PCR was performed using the miRNA qRT-PCR Starter Kit on a 7500 Fast Real-Time PCR system following the manufacturer’s instructions. The relative expression levels of the genes were normalized to that of U6 by using 2 ^−ΔΔCt^.

### Luciferase reporter assays

For reporter assay analyses, 1.2 × 10^4^ cells (HEK293T) in a 96-well plate were transfected with 50 nM miR-222-3p or mimic NC (RiboBio). The cells were then co-transfected with 2 μg/mL of vector with the wild-type or mutant CDKN1B or CDKN1C 3′-UTR. 48 h later, luciferase activity was measured by the Dual-Luciferase^®^ Reporter Assay System (Promega, Madison, WI, USA) according to the manufacturer’s instructions.

### Immunoblotting

Isolated exosome pellet or cultured HA-VSMCs were lysed in RIPA (Radio-Immunoprecipitation Assay) buffer supplemented with complete protease inhibitor cocktail tablets (Roche, Basel, Swiss). Lysates of cells or exosomes were separated by 8–12% SDS-polyacrylamide gels, transferred to PVDF membranes. The membranes were blocked for 2 h in 5% BSA buffer, then incubated with primary antibodies at 4 °C overnight. Protein expression levels were semi-quantitatively analyzed using densitometry analysis.

Anti-CD63 (ab59479), anti-Hsp70 (ab2787) and anti-Alix (ab117600) were purchased from Abcam (Cambridge, MA). Anti-CDKN1B (3686T), anti-CDKN1C (2557T), anti-Cyclin D1 (2922S), anti-MMP2 (13132S), and anti-MMP9 (13667T) antibodies were obtained from CST (Beverly, MA, USA). Anti-β-actin, secondary antibody (HRP-goat anti-mouse IgG and HRP-goat anti-rabbit IgG) were purchased from Weiao (Shanghai, China).

### Histological analysis

For histological analysis, hematoxylin and eosin (H&E), Elastica van Gieson (EVG), immunofluorescence, and standard immunohistochemical staining were performed, according to the standard protocols; six sections taken from the middle portion of each artery, 28 days after the carotid artery injury, were examined; and the neointimal area, medial area, and neointima/media (NI/M) ratio were calculated. Anti-α-SMA (ab124964) were purchased from Abcam (Cambridge, MA). Anti-Ki67 antibody (GB13030-2) and fluorescent secondary antibodies were obtained from Servicebio (Wuhan, China).

### Statistical analysis

All of the results reported here are representative of at least three independent experiments and the data are presented as the mean ± SEM (standard error of mean). All data were first evaluated for normal distribution using the Kolmogorov–Smirnov test. When data were normally distributed and group variances were equal, comparisons between two groups were performed by the Student *t*-test. One-way ANOVA or Two-way ANOVA was used for multiple comparisons between ≥3 groups followed by Tukey’s post-hoc test or Student’s *t*-test when data were normally distributed and group variances were equal. When group data were not normally distributed or if group variances were unequal, the Kruskal–Wallis test followed by the Dunn post-hoc test was used. P values < 0.05 were considered statistically significant. All statistical analyses were performed using GraphPad Prism 5.0 (Graph Pad Prism Software Inc, San Diego, CA, USA) and SPSS software (version 17.0, SPSS Inc., Chicago, IL, USA) for Windows.

## Results

### M1 macrophages contributed to smooth muscle cell functional change

**Fig. 1:**
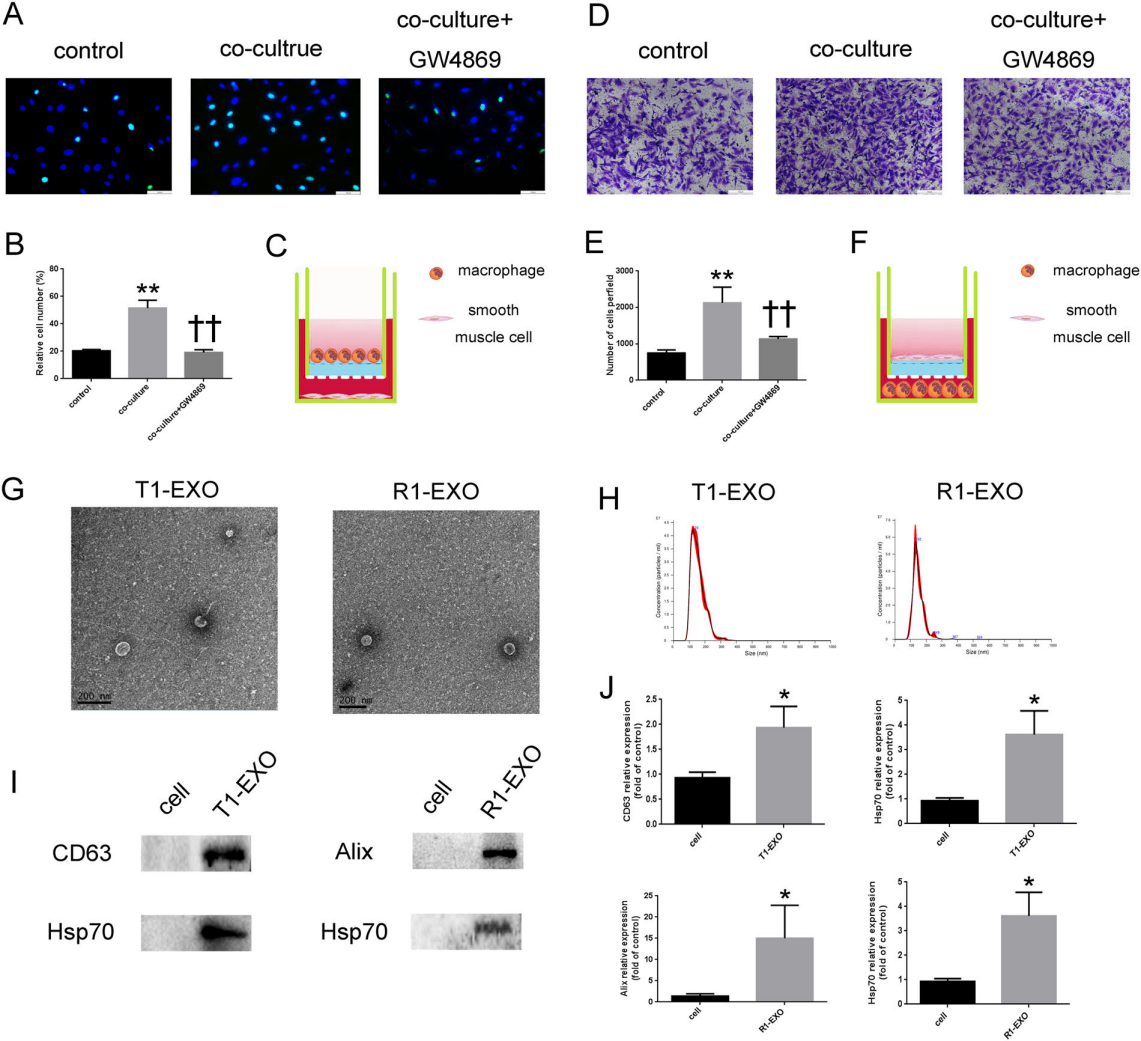
Smooth muscle cell function was affected by M1 macrophages and successful isolation of exosomes derived from M1 macrophages. **a**, **b** M1 macrophages were positively associated with HA-VSMC proliferation. GW4869, a well-known inhibitor of exosome secretion, is used at a concentration of 10 μM to reduce the release of exosome from M1M. *P < 0.05, versus Control group; ^†^*P* < 0.05, versus co-culture group. *n* = 3, each group. **c** Schematic co-culture model for M1M affect VSMCs proliferation. **d**, **e** M1M were positively associated with HA-VSMC migration. GW4869, a well-known inhibitor of exosome secretion, is used at a concentration of 10 μM to reduce the release of exosome from M1M. *P < 0.05, versus Control group; ^†^*P* < 0.05, versus co-culture group. *n* = 3, each group. **f** Schematic co-culture model for M1M affect VSMCs migration. **g** The ultrastructure of R1-EXO and T1-EXO showed typical cup-shaped morphology by transmission electron microscopy. **h** NTA demonstrates the size distribution of T1-EXO and R1-EXO revealing a size peak of 123 and 132 nm, respectively. **i**, **j** The expression of exosomes markers, Alix, Hsp70, and CD63 were confirmed by immunoblotting. A total of 20 μg protein from cell lysis and 20 μg protein from exosomes lysis was loaded into each lane. *P < 0.05, versus Control group. *n* = 3, each group. All data were expressed as mean ± SEM from three individual experiments

To clarify whether M1M were involved in the process of proliferation and migration in SMCs, we co-cultured THP-1 derived M1M with HA-VSMCs in a transwell system, which allowed the transfer of cellular secretions but prevented the transfer of vesicles larger than exosomes and direct cell contact. GW4869, a well-known inhibitor of exosome secretion, was used to reduce the release of exosomes from M1M. EdU staining results showed that HA-VSMCs were significantly activated to proliferate in the presence of M1M secretion,but these effects were abolished in the presence of GW4869 (Fig. 1a–c). Transwell results demonstrated that VSMCs migration was also significantly increased in the cell co-culture system. But these effects were remarkably attenuated after GW4869 treatment (Fig. 1d–f). These data suggested that M1M contributed to smooth muscle cells functional change and exosomes might be involved in the process.

### Successful collection and identification of exosomes derived from M1 macrophages

To further confirm whether M1M-derived exosomes played a key role in the functional change of VSMCs, we chose the THP-1 and RAW264.7 as models for studying M1M-secreted exosomes (named T1-EXO and R1-EXO, respectively) and miRNAs. After macrophages polarization induction, exosomes were purified from conditioned media by ultracentrifugation. As shown in Fig. 1g, exosomes derived from M1M exhibited typical cup-shaped morphology by transmission electron microscopy and a size range of 30–150 nm. To further investigate the size distribution profile of T1-EXOs and R1-EXOs, we performed a size detection using the Nanosight, revealing a size peak of 123 and 132 nm (Fig. 1h). Then the expression of exosome markers, Alix, CD63, and Hsp70, were detected to confirm the identity of the exosomes (Fig. 1i, j). These data indicated a successful isolation of exosomes from M1M.

### Cellular uptake and Endocytic mechanism of exosomes by HA-VSMC cells

**Fig. 2:**
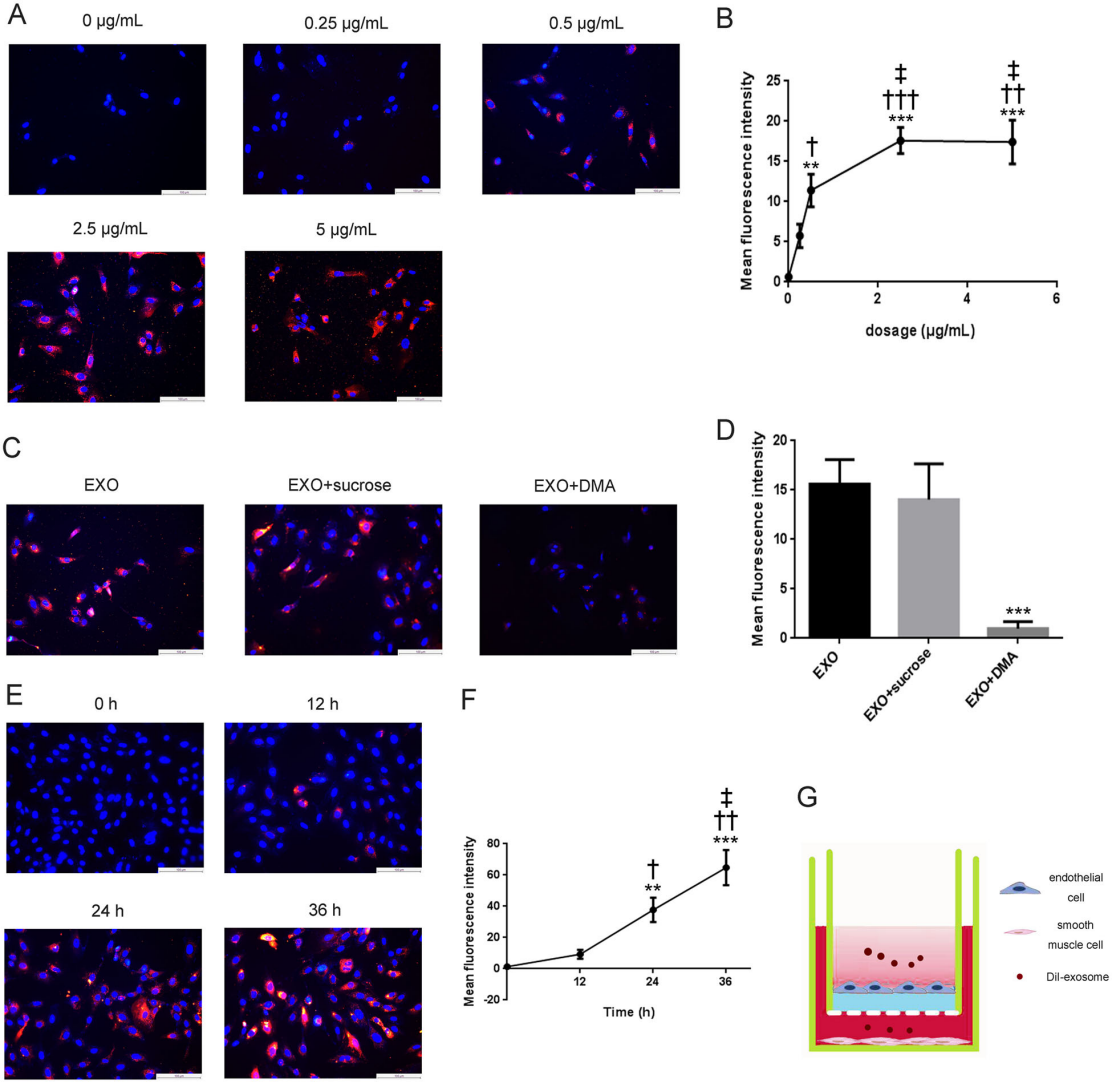
Cellular uptake and endocytic mechanism in HA-VSMC and permeation of T1-EXO across endothelial cell layer. **a**, **b** Representative fluorescent microscopy of HA-VSMCs that were exposed to DiI-labeled exosomes derived from M1M for 6 h. *P < 0.05, versus 0 μg/mL group; ^†^*P* < 0.05, versus 0.25 μg/mL group; ^‡^*P* < 0.05, versus 0.5 μg/mL group. *n* = 3, each group. **c**, **d** Effect of sucrose and DMA on cellular uptake of DiI-labeled T1-EXO in HA-VSMCs. Fluorescence intensity of HA-VSMCs exposed to 2.5 μg exosomes/mL without pre-incubation with any inhibitors representing the maximum internalized amount of DiI-labeled T1-EXO served as a control. *P < 0.05, versus EXO group. *n* = 3, each group. **e**, **f** Representative fluorescent images of HA-VSMC after treatment with DiI-labeled T1-EXO. *P < 0.05, versus 0 h group; ^†^*P* < 0.05, versus 12 h group; ^‡^*P* < 0.05, versus 24 h group. *n* = 3, each group. **g** Schematic diagram of permeation of T1-EXO across endothelial cell monolayer into smooth muscle cells. All data were expressed as mean ± SEM from three individual experiments

To identify whether T1-EXO could be taken up by HA-VSMCs, T1-EXO were labeled with the DiI dye, which has a strong red fluorescence. As highlighted by our fluorescent microscopy images, these exosomes were found to enter VSMCs in a concentration-dependent manner (Fig. 2a, b).

To further investigate the endocytic mechanism of exosomes in HA-VSMCs, cells were pre-incubated with different inhibitory reagents for 30 min. The effects of clathrin-mediated endocytosis on the internalization of exosomes were evaluated using sucrose, a kind of clathrin-coated pits formation blocking agent^24,25^. DMA, a microtubule-disrupting agent, was used to evaluate the effects of macropinocytosis on the internalization of exosomes in VSMCs^10,24,26^. Our results showed that HA-VSMCs pre-incubated with DMA exhibited significant red fluorescence decrease in the cytoplasm. While sucrose did not significantly reduce cellular uptake of exosomes by HA-VSMCs (Fig. 2c, d).

As a cellular communication mediator, exosomes would transport across endothelial monolayer and then permeate into VSMCs. Therefore, we constructed endothelial monolayer and VSMCs co-culture model in this study to investigate the permeation efficacy of exosomes derived from M1M. Interestingly, we noted that exosomes could be uptaken by HA-VSMCs as quick as 12 h after addition and the fluorescence intensity in the cytoplasm of HA-VSMCs was increased in a time-dependent manner (Fig. 2e–g). Taken together, our results indicated that exosomes derived from M1M could be uptaken by HA-VSMC mainly through macropinocytosis endocytic pathway and they had trans-endothelial ability to permeate into smooth muscle cells.

### Potential involvement of miR-222 in T1-EXO in promoting HA-VSMC migration and proliferation

**Fig. 3:**
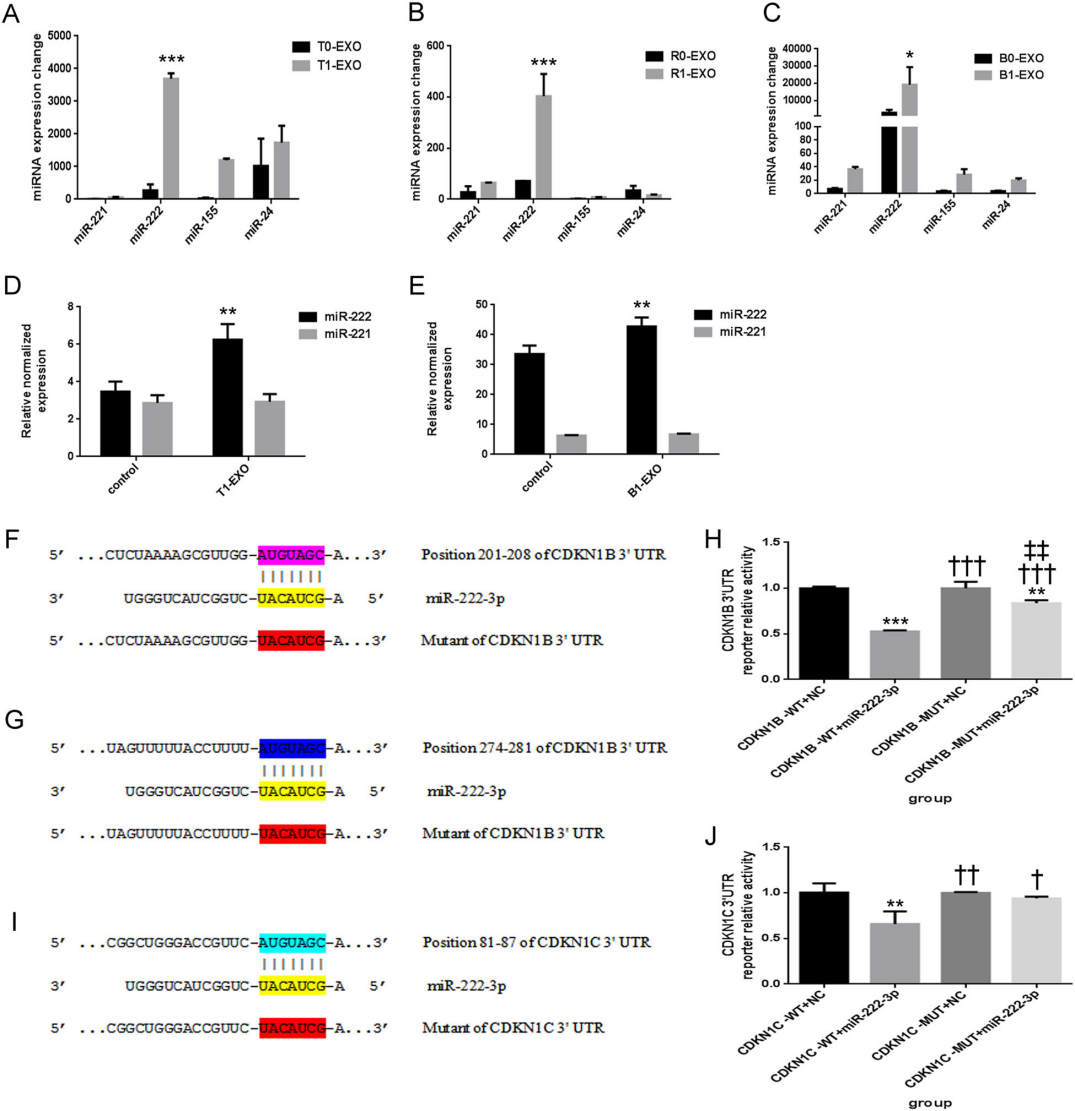
Potential involvement of miR-222 in T1-EXO and direct target genes of miR-222. **a–c** qPCR analysis of principal miRNAs that have been reported to play an important role in VSMCs functional changes. Four miRNAs (miR-221, miR-222, miR-155, and miR-24) were detected in exosomes from M0 macrophages and M1 macrophages, but only miR-222 was most significantly upregulated. *P < 0.05, versus exosomes from M0 macrophages group. *n* = 3, each group. **d**, **e** qPCR analysis of miR-222 expression in HA-VSMCs or primary VSMCs after incubation with T1-EXO or B1-EXO. *P < 0.05, versus control group. *n* = 3, each group. **f**, **g** Schematic representation of the putative binding sites in CDKN1B and CDKN1B mutant mRNAs 3′UTR for miR-222. **h** A luciferase reporter carrying the 3′UTR of CDKN1B (CDKN1B-WT) or mutant CDKN1B (CDKN1B-MUT) was introduced into 293T cells along with negative miR-control (NC) or miR-222-3p. Transfected cells were subjected to serum starvation for 48 h, and cell lysates were subjected to luciferase activity assay. *P < 0.05, versus CDKN1B-WT+NC group; ^†^*P* < 0.05, versus CDKN1B-WT+miR-222-3p group; ^‡^*P* < 0.05, versus CDKN1B-MUT+NC group. *n* = 3, each group. **i** Schematic representation of the putative binding sites in CDKN1C and CDKN1C mutant mRNAs 3′UTR for miR-222. **j** A luciferase reporter carrying the 3′UTR of CDKN1C (CDKN1C-WT) or mutant CDKN1C (CDKN1C-MUT) was introduced into 293T cells along with negative miR-control (NC) or miR-222-3p. Transfected cells were subjected to serum starvation for 48 h, and cell lysates were subjected to luciferase activity assay. *P < 0.05, versus CDKN1C-WT+NC group; ^†^*P* < 0.05, versus CDKN1C-WT+miR-222-3p group. *n* = 3, each group. All data were expressed as mean ± SEM from three individual experiments

Next, we applied microRNA array analyses to determine the expression change of four exosome-associated microRNAs that could induce functional change in VSMCs (miR-221, miR-222, miR-155, and miR-24) before or after macrophage polarization. Exosomes isolated from THP-1 derived M1 or M0 macrophages were named as T1-EXO or T0-EXO. Exosomes isolated from RAW264.7-derived M1 or M0 macrophages were named as R1-EXO or R0-EXO. Exosomes isolated from BMDM derived M1 or M0 macrophages were named as B1-EXO or B0-EXO. As shown in Fig. 3a–c, miR-222 expression levels showed the most significant increase among these microRNAs in exosomes after macrophage polarization. In order to explore whether miR-222 could be delivered from exosomes to VSMCs, we evaluated miR-222 expression changes in HA-VSMCs and primary VSMCs. After 24 h incubation, miR-222 expression exhibited a remarkable increase in the cells (Fig. 3d, e).

To determine the mechanism by which miR-222 regulate VSMCs function, we performed miRNA target search using biological intelligence analyses and found 3′UTR of CDKN1B and CDKN1C containing the highly conserved putative miR-222 binding sites (Fig. 3f, g, i). These data indicated that miR-222 in exosomes derived from M1M might be the activator of VSMCs functional changes.

To further clarify whether or not CDKN1B and CDKN1C were the direct targets of miR-222 during VSMCs functional change, luciferase reporter assays were performed. As expected, transfection with miR-222-3p significantly repressed luciferase activity in wild-type 3′-UTR groups, while no suppression of activity was observed with respect to the mutant ones (Fig. 3h, j). Collectively, our results suggested that CDKN1B and CDKN1C were direct targets of miR-222.

### T1-EXO and miR-222 enhanced smooth muscle cells proliferation

**Fig. 4:**
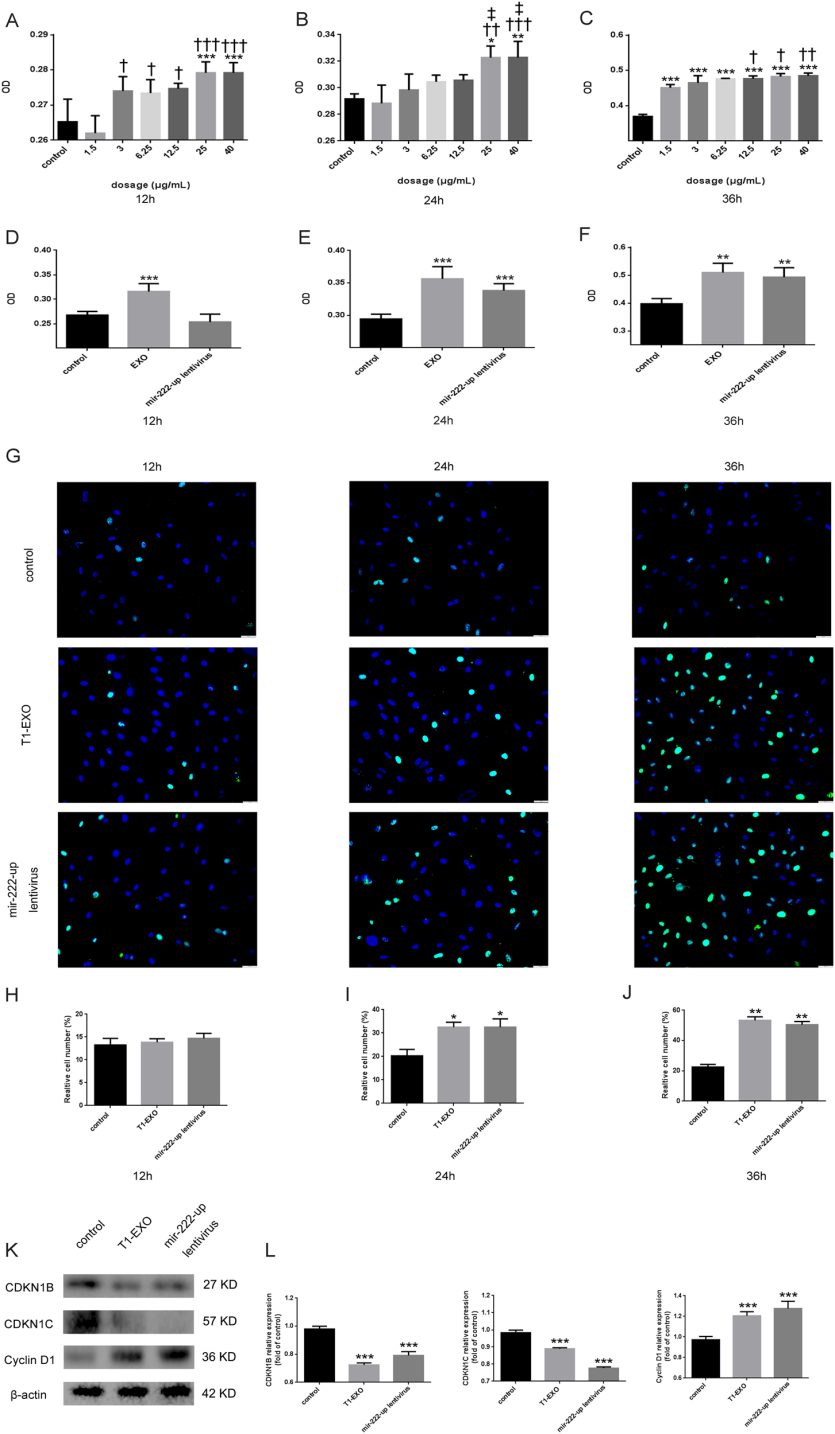
T1-EXO and miR-222 promoted HA-VSMCs proliferation. **a–c** The effect of different concentrations of T1-EXO on HA-VSMCs proliferation was detected by CCK8. *P < 0.05, versus control group; ^†^*P* < 0.05, versus 1.5 μg/mL group; ^‡^*P* < 0.05, versus 3 μg/mL group. *n* = 5, each group. **d–f** The pro-proliferative effect of optimal concentration of T1-EXO and mir-222-up lentivirus in HA-VSMCs was compared by CCK8. *P < 0.05, versus control group. *n* = 5, each group. **g–j** Representative EdU-stained VSMC photomicrographs from different groups. Note: green color is EdU positive cell; blue color is nucleus stained by Hoechst 33342. *P < 0.05, versus control group. *n* = 3, each group. **k**, **l** T1-EXO and miR-222 modulates HA-VSMC proliferation by regulating proteins that control cell cycle. T1-EXO and miR-222 down-regulated the expression of CDKN1B and CDKN1C but upregulated the expression of Cyclin D1 in HA-VSMC. β-actin expressions served as internal control. The control group was set to 1. *P < 0.05, versus control group. *n* = 3, each group. All data were expressed as mean ± SEM from three individual experiments

Proliferation was evaluated following the stimulation of HA-VSMCs with various doses of T1-EXO. CCK8 assay clearly showed that T1-EXO significantly promoted HA-VSMCs proliferation in a dose-dependent manner (Fig. 4a–c). At a concentration of 25 μg exosomes/mL, VSMCs cultured with T1-EXOs showed greater proliferation than those incubated with other doses of exosomes, so we chose 25 μg/mL as an optimal concentration for future experiments. Then we conducted the pro-proliferative efficacy comparison between T1-EXO and miR-222. As expected, both T1-EXO and miR-222 promoted proliferation for HA-VSMCs in a time-dependent manner (Fig. 4d–f). Consistent with these findings, similar results were also observed regarding the better effects in promoting HA-VSMC proliferation after T1-EXO or miR-222 treatment than control in EdU incorporation assay (Fig. 4g–j). Therefore, These results may lead us to note that T1-EXO exhibits its functions via miR-222.

Since CDKN1B and CDKN1C have shown to be involved in cell-cycle regulation and Cyclin D1 is a key regulator in cell-cycle progression, the expression levels of these three cell-cycle-associated proteins were evaluated by western blotting in the presence of the T1-EXO or mir-222-up lentivirus. Our results showed that the expression of CDKN1B and CDKN1C were significantly decreased, while Cyclin D1 was remarkably upregulated. Collectively, these findings demonstrated that T1-EXO and miR-222 exhibited pro-proliferative functions by regulating cell cycle associated proteins in VSMCs.

### Both T1-EXO and miR-222 promoted smooth muscle cells migration

**Fig. 5:**
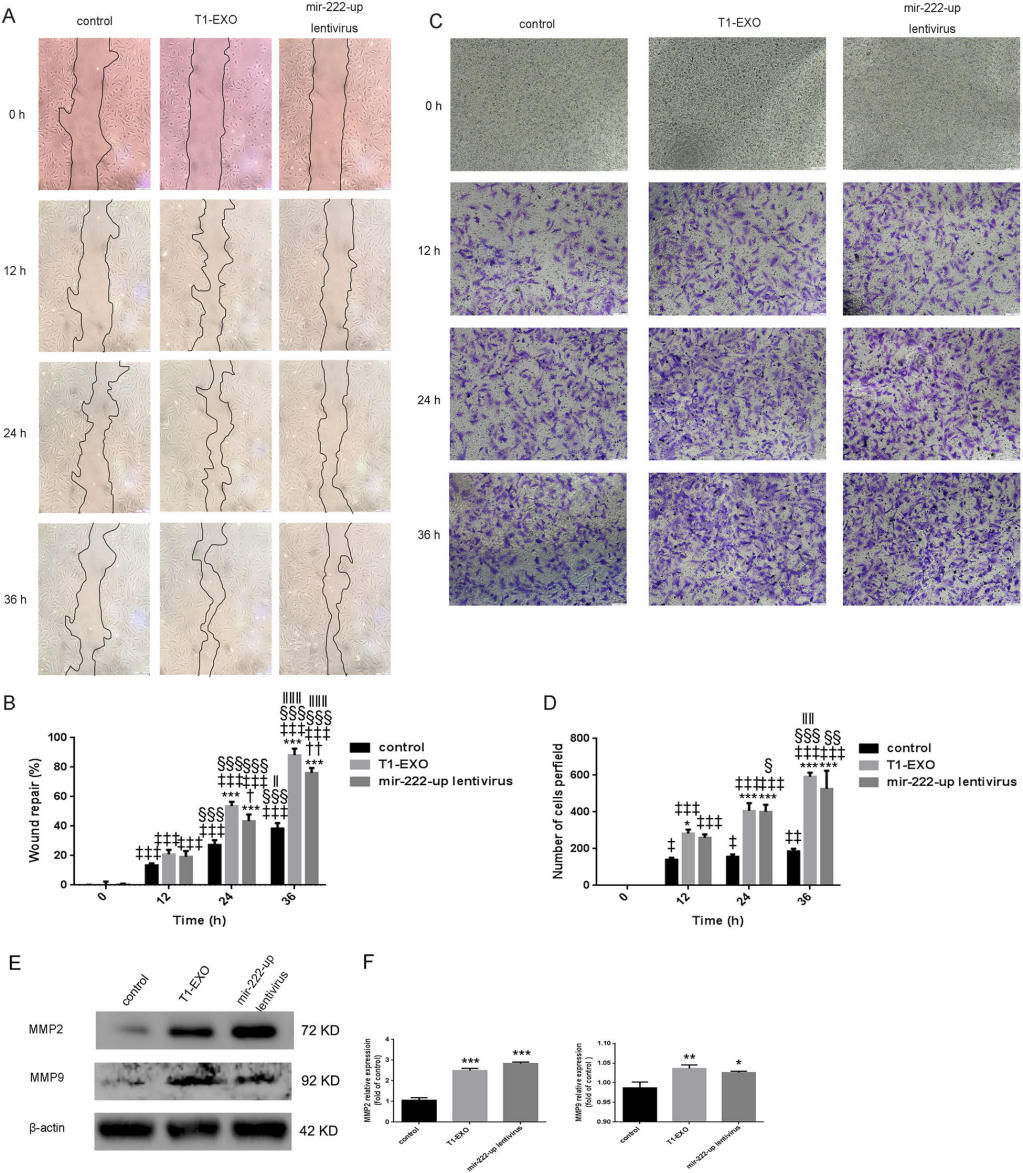
T1-EXO and miR-222 enhanced HA-VSMC migration. **a** Quiescent scratched HA-VSMCs were treated or untreated with T1-EXO (25 μg/mL) in FBS-free medium for 36 h. VSMCs infected with mir-222-up lentivirus served as a positive control. Phase-contrast microscopy images showing the migratory capacity of VSMCs under different treatment. **b** Diagram showed quantification of cell migration rate over time. *P < 0.05, versus control group; ^†^*P* < 0.05, versus T1-EXO group; ^‡^*P* < 0.05, versus 0 h group; ^§^P < 0.05, versus 12 h; ^‖^*P* < 0.05, versus 24 h group. *n* = 3, each group. **c** A transwell assay was performed to assess HA-VSMCs migration. **d** Diagram showed quantification of migrated cells through transwell chamber. *P < 0.05, versus control group; ^‡^*P* < 0.05, versus 0 h group; ^§^*P* < 0.05, versus 12 h; ^‖^*P* < 0.05, versus 24 h group. *n* = 3, each group. **e**, **f** T1-EXO and miR-222 improved the expression of proteins involved in smooth muscle cell migration: MMP2 and MMP9. β-actin expressions served as internal control. The control group was set to 1. *P < 0.05, versus control group. *n* = 3, each group. All data were expressed as mean ± SEM from three individual experiments

To demonstrate whether T1-EXO and miR-222 could improve migratory ability in recipient cells, HA-VSMCs were subjected to wound healing and Transwell function experiments. Both exosomes and miR-222 were found to enhance VSMCs migration in a time-dependent manner (Fig. 2a), and T1-EXO even exhibited a better pro-migratory ability at any time point compared with control and miR-222 (Fig. 5a, b). In line with above findings, Transwell assays further confirmed the biological effects of T1-EXO and miR-222 on HA-VSMCs migration (Fig. 5c, d). To interpret the underlying molecular mechanisms, western blotting was applied. We observed a positive correlation between the expression levels of MMP2 and MMP9 in VSMCs and T1-EXO or miR-222 treatment (Fig. 5e, f). Taken together, all these data addressed this issue and indicated that T1-EXO regulated VSMCs function through delivering miR-222 into VSMCs.

### R1-EXO aggravated neointimal hyperplasia following carotid artery injuries and 2′OMe-miR-222 abolished these effects

To validate the in vitro data of M1M-derived exosomes, we investigated the role of R1-EXO in neointimal hyperplasia using two carotid artery injury models. After carotid artery ligation injury and wire injury models were established, mice were treated with pluronic gel F-127 preloaded with R1-EXO or oligonucleotides.

**Fig. 6:**
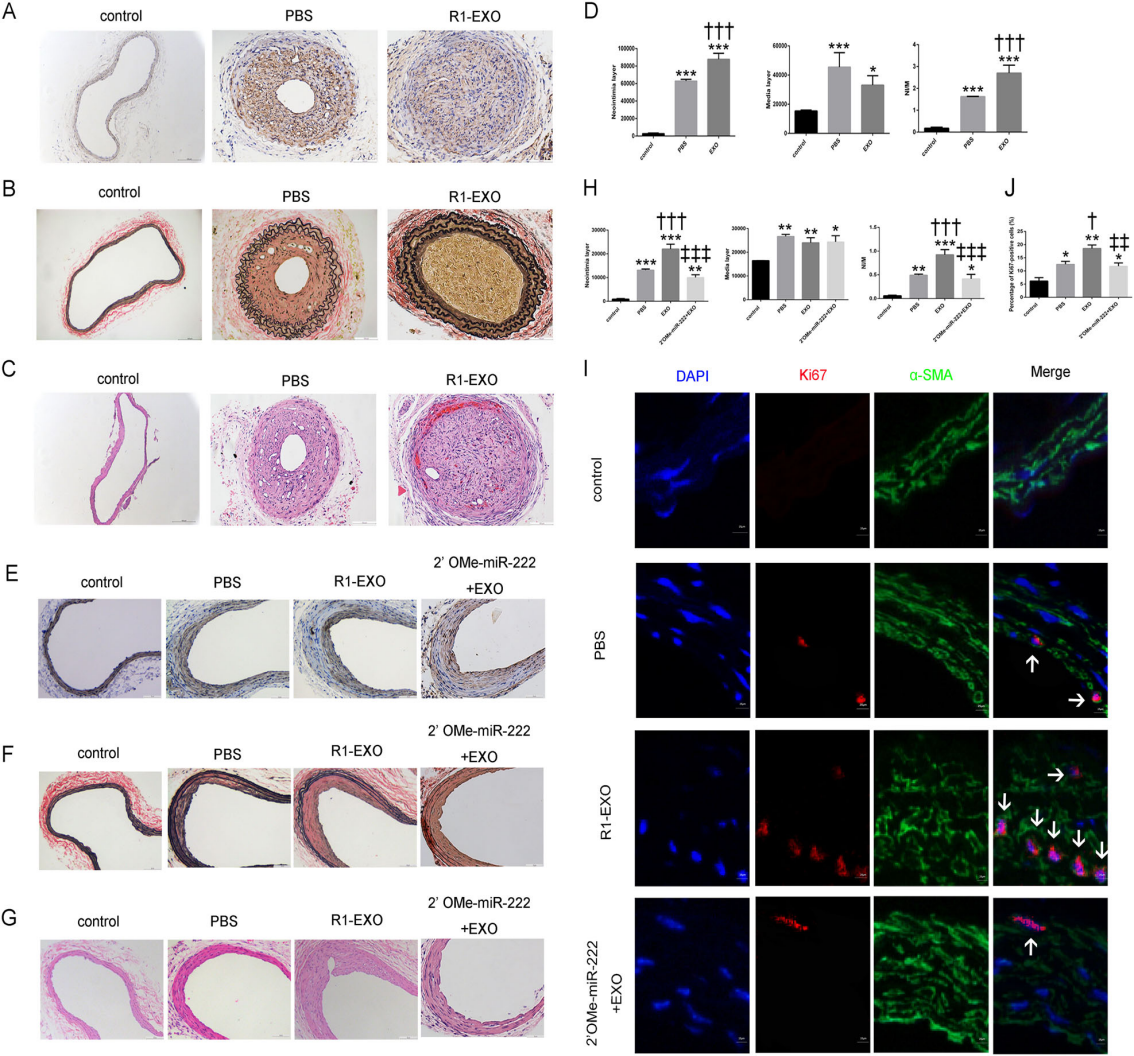
R1-EXO aggravated neointima formation following carotid artery ligation injury and wire injury but 2′OMe-miR-222 abolished these effects. **a** Neointima smooth muscle cell infiltration was determined by immunohistochemical staining against α-SMA. **b**, **c** Neointima formation was determined on hematoxylin and eosin-stained and Elastica van Gieson-stained cross sections of carotid arteries 28 days after vascular ligation injury. **d** Bar graphs showed quantification of neointima area, media area and neointima/media ratio under different treatment. *P < 0.05, versus control group; ^†^*P* < 0.05, versus PBS group. *n* = 5, each group. **e** 28 days after injury, neointima smooth muscle cell infiltration was determined by immunohistochemical staining against α-SMA. **f**, **g** Neointima formation was determined on hematoxylin and eosin-stained and Elastica van Gieson-stained cross sections of carotid arteries 28 days after vascular wire injury. **h** Bar graphs showed quantification of neointima area, media area and neointima/media ratio under different treatment. *P < 0.05, versus control group; ^†^*P* < 0.05, versus PBS group; ^‡^*P* < 0.05, versus EXO group. *n* = 5, each group. **i**, **j** Representative Immunofluorescence staining for Ki67 after wire injury. Arrows indicated Ki67-positive smooth muscle cells. Percentage of Ki67-positive cells of carotid arteries of mice from either the control groups or different treatment groups. *P < 0.05, versus control group; ^†^*P* < 0.05, versus PBS group; ^‡^*P* < 0.05, versus EXO group. *n* = 5, each group. All data were expressed as mean ± SEM from three individual experiments

Four weeks after surgery, immunohistochemical staining with monoclonal antibodies against α-SMA on frozen sections demonstrated the presence of abundant VSMCs in vessel lesions (Fig. 6a). The effect of R1-EXO on neointima formation was further evaluated by H&E and Elastica van Gieson staining on vessel lesions. Treatment of exosomes derived from M1M caused a substantial increase in neointimal layer thickness and neointima/media ratio compared to arteries treated with PBS. However, no significant changes were observed in the media layer thickness between the PBS and R1-EXO group (Fig. 6b, c).

In order to further confirm the specific contribution of miR-222 in M1M-secreted exosomes on neointima formation and to secure the restenosis aggravation, a miR-222 inhibitor treatment (2′OMe-miR-222) was introduced on wire-injured carotid arteries. The results showed 2′OMe-miR-222+R1-EXO treatment remarkably withdrew the malignant phenotypes induced by R1-EXO (Fig. 6e–h). More importantly, with treatment of 2′OMe-miR-222+R1-EXO, the NI/M ratio was dramatically decreased as half of the R1-EXO group, suggesting that 2′OMe-miR-222 repressed the cell accumulation induced by treatment with R1-EXO in vivo (Fig. 6h).

In addition, Ki67 staining was conducted to determine whether above treatments affect VSMCs proliferative status in vivo. Ki67 is a nuclear antigen expressed in S, G2, M, and post-mitotic G1 phases of the cell cycle but not in resting cells in G0 and thus indicative of proliferation. As shown in Fig. 6i–j, R1-EXO remarkably promoted the percentage of Ki67-positive cells in neointima, while 2′OMe-miR-222 significantly suppressed the effect of R1-EXO on the growth of VSMCs. To conclude, our results indicated that exosomes derived from M1M could aggravate neointimal hyperplasia following carotid artery injuries and these effects could be partially abolished in the presence of miR-222 inhibitor.

## Discussion

**Fig. 7:**
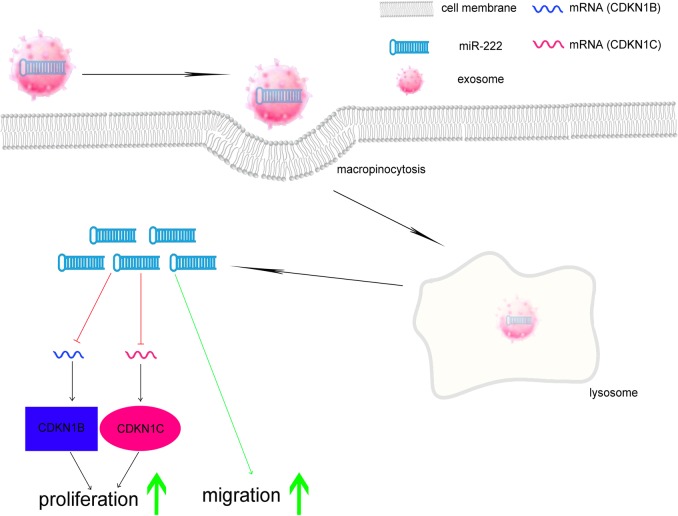
Schematic diagram of M1 macrophage exosomes intracellular stimulation in VSMCs

In this study, we demonstrated that exosomes derived from M1M were involved in neointimal hyperplasia by targeting CDKN1B and CDKN1C through transferring miR-222 to recipient cells. To our knowledge, this is the first study to show exosome-mediated intercellular communication between M1 macrophages and smooth muscle cells, resulting in a malignant pathogenesis after vascular injury (Fig. 7).

The cellular composition of neointima greatly affects the success rate of stent implantation after PCI^27^. Previous studies showed that macrophages are likely to play a central role in inducing vascular smooth muscle cell accumulation within the neointima by releasing cytokines in the microenvironment^27^. But study focusing on extracellular vesicles-based intercellular communication between macrophages and VSMCs is still rare. One representative example is that macrophage foam cell-derived extracellular vesicles could promote migration and adhesion of vascular smooth muscle cells^28^. Niu et al. compared the effect of extracellular vesicles released by foam cell and normal macrophages on VSMCs function. With the help of proteomic experiments they identified 599 proteins and showed that these proteins may act on VSMCs through two different pathways. While in our study, we found M1M could have a deep impact on the functional change of VSMCs through transferring exosomes.

Previous studies have shown that HA-VSMCs can take up exosomes^28^. In order to reveal the endocytic mechanisms in HA-VSMCs, we labeled exosomes isolated from conditioned medium with DiI. Our results showed that not only could exosomes be uptaken by HA-VSMCs but the major endocytic mechanism was macropinocytosis. Endocytosis, which occurs in most cells as pinocytosis, has at least four basic mechanisms: clathrin-mediated endocytosis, caveolae-mediated endocytosis, macropinocytosis and clathrin and caveolae-independent endocytosis. Recent studies have shown that cellular uptake of extracellular vesicles is mainly mediated by clathrin-independent endocytosis and macropinocytosis^24^. As shown in Fig. 2c, DMA, a microtubule-disrupting agent, could significantly decrease the cellular uptake of T1-EXO in HA-VSMCs, which indicated the major internalization pathway of T1-EXO by HA-VSMC occurred through macropinocytosis. On the other hand, sucrose, a clathrin-coated pits formation blocking agent, did not influence the cellular uptake of T1-EXO evidently, which suggested the minimal contribution of clathrin-dependent endocytosis to the internalization of T1-EXO by HA-VSMCs. All these data suggested that the major endocytic mechanisms of M1M-derived exosomes by smooth muscle cells is clathrin-independent endocytosis and macropinocytosis, which is consistent with the previous reports^24^.

miRNAs, as crucial mediators for the benefits of exosomes, can provide sustained therapeutic effect and fundamental alterations of the local microenvironment. However, little is known about which miRNAs are associated with M1M-induced VSMCs functional change. As a different kind of miRNAs have been proved to be enriched in exosomes released by M1M^29,30^, it seems reasonable to speculate that the potentially effective miRNAs as connectors should be abundant in M1M-exosomes. Therefore, differentially expressed miRNAs in macrophage derived exosomes were detected by qRT-PCR, suggesting a possible connection between the increases of these miRNAs and VSMCs functional change. Among these miRNAs, only miR-222 levels in M1M-exosomes were significantly greater than that in M0M-exosomes. On the other hand, a recent study reported by Seeley et al. also noticed that prolonged stimulation with lipopolysaccharide in macrophages will induce a huge increase of miR-222 in bone-marrow-derived macrophages^31^. Moreover, we showed that introduction of 2′OMe-miR-222 significantly abolished M1M-EXOs effects in enhancing neointimal lesion formation. All these compelling evidence suggested that we successfully built a bridge between M1M exosomal microRNAs and VSMCs functional change and miR-222 was the specific contributor in M1M-derived exosomes.

CDKN1B and CDKN1C are two members of cyclin inhibitory proteins family. CyclinA-CDK2, cyclinD1-CDK2, and cyclinE-CDK2 are complexes operate at the G1 to S transition. The cyclin inhibitory proteins could bind to and prevent the activation of cyclin-CDK complexes, and thus controls the cell-cycle progression at G1^32^. In our study we found that the ability of VSMCs proliferation was drastically increased after T1-EXO treatment, which might be due to the down-regulating of CDKN1B and CDKN1C by miR-222 in cell-cycle progression, and Ki67 staining results in vivo further confirmed our hypothesis that M1M-EXO were more likely to promote VSMCs to pass through G1/S cell-cycle checkpoint and to stay in proliferative status.

Finally, we performed in vivo experiments to verify the in vitro results. Even though penetration efficiency of exsosomes through the vascular endothelium in vivo was not evaluated in our study, there have been a number of studies to show that nanoparticles or even naïve macrophage exosomes could be developed as potential drug delivery systems for enhancing the endothelial barrier penetration and improving the drug accumulation in the lower tissues. Yuan et al. labeled macrophage exosomes with radioactive element ^125^I and injected these exosomes through the tail vein. Exosomes could penetrate blood–brain barrier (BBB) via ICAM-1 receptor-mediated transcytosis^33^. Dual-targeted nanoparticles could also improve the BBB penetration via GLUT-mediated transcytosis and promote the drug accumulation in the glioma^34^. Our endothelial-across results suggested that the exosomes derived from M1M could transport across endothelial monolayer and permeate into VSMCs. Furthermore, pluronic gel F-127, a well acceptable bioengineering material, was applied to simulate sustained release of exosomes in vivo. Amanda K. A. Silva et al. has reported pluronic gel F-127 preloaded with extracellular vesicles derived from adipose tissue-derived stromal cells (ASCs) could effectively retain EVs in the entire fistula tract and heal the esophageal fistula^35^.

In conclusion, the results presented here demonstrate that miR-222 in exosomes derived from M1M is essential during restenosis formation. To the best of our knowledge, this is the first study to demonstrate the regulation of VSMCs function by an exosomal miRNA during neointimal hyperplasia. Our results thus not only provide new insights into the function regulation of VSMCs by exosomal miRNAs, but also indicate that M1 macrophage exosomal miR-222 may exhibit potential as a therapeutic drug target.

## References

[1] Shishido K (2016). Effects of low endothelial shear stress after stent implantation on subsequent neointimal hyperplasia and clinical outcomes in humans. J. Am. Heart Assoc..

[2] Rosenbaum MA, Miyazaki K, Graham LM (2012). Hypercholesterolemia and oxidative stress inhibit endothelial cell healing after arterial injury. J. Vasc. Surg..

[3] Chang SH (2018). Propylthiouracil-coated biodegradable polymer inhibited neointimal formation and enhanced re-endothelialization after vascular injury. Int. J. Nanomed..

[4] Parks BW, Lusis AJ (2013). Macrophage accumulation in atherosclerosis. N. Engl. J. Med..

[5] Moore KJ, Tabas I (2011). Macrophages in the pathogenesis of atherosclerosis. Cell.

[6] Lavin B (2014). Nitric oxide prevents aortic neointimal hyperplasia by controlling macrophage polarization. Arterioscl. Throm. Vas..

[7] Valadi H (2007). Exosome-mediated transfer of mRNAs and microRNAs is a novel mechanism of genetic exchange between cells. Nat. Cell Biol..

[8] Raposo G, Stoorvogel W (2013). Extracellular vesicles: exosomes, microvesicles, and friends. J. Cell Biol..

[9] Liu X, Cheng Y, Yang J, Xu L, Zhang C (2012). Cell-specific effects of miR-221/222 in vessels: molecular mechanism and therapeutic application. J. Mol. Cell. Cardiol..

[10] Liu X (2009). A necessary role of miR-221 and miR-222 in vascular smooth muscle cell proliferation and neointimal hyperplasia. Circ. Res..

[11] Mohr A, Mott J (2015). Overview of microRNA biology. Semin. Liver Dis..

[12] Chistiakov DA, Orekhov AN, Bobryshev YV (2015). Extracellular vesicles and atherosclerotic disease. Cell. Mol. Life Sci..

[13] Wei Y, Nazari-Jahantigh M, Neth P, Weber C, Schober A (2013). MicroRNA-126, -145, and -155: a therapeutic triad in atherosclerosis?. Arterioscl. Throm. Vas..

[14] McDonald RA (2013). miRNA-21 is dysregulated in response to vein grafting in multiple models and genetic ablation in mice attenuates neointima formation. Eur. Heart J..

[15] Zhang R (2014). Tongxinluo inhibits vascular inflammation and neointimal hyperplasia through blockade of the positive feedback loop between miR-155 and TNF-α. Am. J. Physiol. Heart C.

[16] Chistiakov DA, Sobenin IA, Orekhov AN, Bobryshev YV (2015). Human miR-221/222 in physiological and atherosclerotic vascular remodeling. Biomed. Res. Int..

[17] Xu Y (2017). MicroRNA-222 promotes the proliferation of pulmonary arterial smooth muscle cells by targeting P27 and TIMP3. Cell. Physiol. Biochem..

[18] Perry, M. M., Baker, J. E., Gibeon, D. S., Adcock, I. M. & Chung, K. F. Airway smooth muscle hyperproliferation is regulated by microRNA-221 in severe asthma. *Am. J. Resp. Cell. Mol*. **50**, 7–17 (2013). 10.1165/rcmb.2013-0067OC PMC3930931 23944957

[19] Wang H (2016). Transplantation of EPCs overexpressing PDGFR-β promotes vascular repair in the early phase after vascular injury. BMC Cardiovasc. Disord..

[20] Ji R (2007). MicroRNA expression signature and antisense-mediated depletion reveal an essential role of microRNA in vascular neointimal lesion formation. Circ. Res..

[21] Wang X (2016). MicroRNA-221 sponge therapy attenuates neointimal hyperplasia and improves blood flows in vein grafts. Int. J. Cardiol..

[22] Wang X (2017). Adenovirus-mediated gene transfer of microRNA-21 sponge inhibits neointimal hyperplasia in rat vein grafts. Int. J. Biol. Sci..

[23] Zhou W (2014). Cancer-secreted miR-105 destroys vascular endothelial barriers to promote metastasis. Cancer Cell..

[24] Costa Verdera H, Gitz-Francois JJ, Schiffelers RM, Vader P (2017). Cellular uptake of extracellular vesicles is mediated by clathrin-independent endocytosis and macropinocytosis. J. Control Release..

[25] Tahara K (2009). Improved cellular uptake of chitosan-modified PLGA nanospheres by A549 cells. Int. J. Pharmaceut..

[26] Nam HY (2009). Cellular uptake mechanism and intracellular fate of hydrophobically modified glycol chitosan nanoparticles. J. Control Release..

[27] Chaabane C, Otsuka F, Virmani R, Bochaton-Piallat ML (2013). Biological responses in stented arteries. Cardiovasc. Res..

[28] Niu C (2016). Macrophage foam cell–derived extracellular vesicles promote vascular smooth muscle cell migration and adhesion. J. Am. Heart Assoc..

[29] McDonald MK (2014). Functional significance of macrophage-derived exosomes in inflammation and pain. Pain.

[30] Hui WW (2018). Salmonella enterica Serovar Typhimurium alters the extracellular proteome of macrophages and leads to the production of proinflammatory exosomes. Infect. Immun..

[31] Seeley JJ (2018). Induction of innate immune memory via microRNA targeting of chromatin remodelling factors. Nature.

[32] Konigsberg R (2008). Cell cycle dysregulation influences survival in high risk breast cancer patients. Cancer Invest..

[33] Yuan D (2017). Macrophage exosomes as natural nanocarriers for protein delivery to inflamed brain. Biomaterials.

[34] Jiang X (2014). Nanoparticles of 2-deoxy-d-glucose functionalized poly(ethylene glycol)-co-poly(trimethylene carbonate) for dual-targeted drug delivery in glioma treatment. Biomaterials.

[35] Silva AKA (2018). Thermoresponsive gel embedded with adipose stem-cell-derived extracellular vesicles promotes esophageal fistula healing in a thermo-actuated delivery strategy. ACS Nano.

